# Post-incident review after restraint in mental health care -a potential for knowledge development, recovery promotion and restraint prevention. A scoping review

**DOI:** 10.1186/s12913-019-4060-y

**Published:** 2019-04-23

**Authors:** Unn Elisabeth Hammervold, Reidun Norvoll, Randi W. Aas, Hildegunn Sagvaag

**Affiliations:** 1 Department of Public Health, Faculty of Health Sciences, University of Stavanger, NO-4036 Stavanger, Norway; 2 Work Research Institute, Oslo Metropolitan University, Oslo, Norway; 3 Department of Occupational Therapy, Prosthetics and Orthotics, Faculty of Health Sciences, Oslo Metropolitan University, Oslo, Norway

**Keywords:** Debriefing, Post-incident review, Restraints, Mental, Restraint reduction, Recovery-oriented care, Reflection

## Abstract

**Background:**

Use of physical restraint is a common practice in mental healthcare, but is controversial due to risk of physical and psychological harm to patients and creating ethical dilemmas for care providers. Post-incident review (PIR), that involve patient and care providers after restraints, have been deployed to prevent harm and to reduce restraint use. However, this intervention has an unclear scientific knowledge base. Thus, the aim of this scoping review was to explore the current knowledge of PIR and to assess to what extent PIR can minimize restraint-related use and harm, support care providers in handling professional and ethical dilemmas, and improve the quality of care in mental healthcare.

**Methods:**

Systematic searches in the MEDLINE, PsychInfo, Cinahl, Sociological Abstracts and Web of Science databases were carried out. The search terms were derived from the population, intervention and settings.

**Results:**

Twelve studies were included, six quantitative, four qualitative and two mixed methods. The studies were from Sweden, United Kingdom, Canada and United States. The studies’ design and quality varied, and PIR s’ were conducted differently. Five studies explored PIR s’ as a separate intervention after restraint use, in the other studies, PIR s’ were described as one of several components in restraint reduction programs. Outcomes seemed promising, but no significant outcome were related to using PIR alone. Patients and care providers reported PIR to: 1) be an opportunity to review restraint events, they would not have had otherwise, and 2) promote patients’ personal recovery processes, and 3) stimulate professional reflection on organizational development and care.

**Conclusion:**

Scientific literature directly addressing PIR s’ after restraint use is lacking. However, results indicate that PIR may contribute to more professional and ethical practice regarding restraint promotion and the way restraint is executed. The practice of PIR varied, so a specific manual cannot be recommended. More research on PIR use and consequences is needed, especially PIR’s potential to contribute to restraint prevention in mental healthcare.

## Background

Restraint is frequently used in mental healthcare in western countries, despite the lack of studies supporting the practice [1, 2]. Restraint is defined as mechanical or physical action, often using straps, belts or other equipment, intended as a last resort to hold patients in order to prevent self-injury, injuries to others, or significant damage to the environment [3]. Laws related to mental health state that the ethical principles of “proportionality” and “purposefulness” must be considered when restraint is used [3]. The principle of proportionality requires that the level of coercive measures is restricted to what is least required for that patient and that situation, and the principle of purposefulness means that coercive measures can only be used when clearly specified reasons have been stated in advance. Furthermore, the patient’s needs and preferences must be taken into consideration and supported by evidence.

The use of restraint is controversial due to the possible negative consequences, including infringement of patients’ autonomy and liberty and the risk of physical and psychological harm to patients and care providers [4–6].

Health care providers should base their practice on respect for fundamental human rights, preserve patients’ integrity and dignity and treat them with care and respect [7, 8].

Cases in which restraint use seems inevitable can challenge this position as ethical principles may conflict with each other. For example, the principle of autonomy may conflict with the principle of inflicting harm (maleficence) in a case where a patient may cause physical harm to him or herself or to others [9]. Thus, ethical and professional imperatives point towards developing reflexive practices aimed at avoiding unnecessary restraint, improving the execution of restraint and helping patients maintain hope and identity during crises [10].

Despite the widespread use of restraint and the associated risks, few studies examine restraint from the perspectives of care and treatment planning. Restraint use is, therefore “an area that begs for research into alternative methods of assessment, caregiving, and treatment planning” ([7], p.11).

Internationally, a growing literature supports implementation of different strategies to reduce both seclusion and restraint (S/R) [11, 12]. For example, to prevent S/R, Huckshorn recommends implementing six core strategies in care environments, based on the principles of recovery: 1) workforce development 2) rigorous debriefing 3) leadership in organisational changes 4) use of data to inform practice 5) use of S/R prevention tools and 6) full inclusion of patients and families [13, 14]. Studies on the outcomes of programmes using these core strategies seem to offer promising results for S/R reduction in mental healthcare [11, 12, 15].

However, it is difficult to assess how much different interventions contribute individually to these supposedly promising results.

One of the core strategies - rigorous debriefing, has been demanded from patients and care providers after restraint incidents for several years [16–19]. Debriefing was originally a procedure used with ambulance personnel after exposure to traumatic situations in their work and was later expanded for use as an early intervention protocol for individuals exposed to a wider range of potentially traumatic events. Due to conceptual confusions and methodological issues, experts have not reached consensus on the value of debriefing [20].

William Fisher [21] however, described two main varieties of debriefing after critical incidents in mental healthcare: 1) debriefing with care providers alone, in post-incident analysis aimed at evaluating what could have been done differently and making short-term plans to avoid repeating restraint use; 2) debriefing for patients and care providers together, consisting of a detailed behaviour analysis of the events preceding restraint use by both parties. Due to the demands of user participation in mental healthcare and national guidelines on debriefing that include both patients and care providers, this review considers the later type of debriefing. Among the many terms used to refer to interventions after restraint are: debriefing procedures, post-event discussion and post-event analysis [22]. However, we have adopted the concept of post-incident reviews (PIR) used by Bonner and Wellmann [23], with the acronym “PIR”.

PIR may be a promising intervention for care planning and S/R reduction in mental healthcare. On this basis, several countries have formalised the use of PIR s’ for patients and care providers together [12]. However, the knowledge base of this requirement is vague, and there seems to be a lack of systematically-summarised knowledge on both the various PIR procedures available and an evaluation of their benefits and dilemmas in patient treatment [22]. This situation creates a need for state of the art of existing knowledge. The aim of this scoping review is to explore knowledge of PIR after restrains in the scientific literature and to assess to what extent can PIR s’ minimise restraint-related patient harm, support care providers in handling professional and ethical dilemmas and improve the quality of care in mental healthcare. More specifically, we ask: (1) How are PIR s’ defined and described? (2) How are PIR s’ conducted in practice, and what are possible variations in PIR use? (3) What are patients’ and care providers’ experiences of PIR? Finally, the question of what are the implications of reviewing the use of PIR as a tool that might benefit both patients and care providers is discussed by drawing on a recovery-oriented framework [10] and the humanising care approach to nursing and ethics [24]. This approach is chosen because of its potential to mitigate consequences like retraumatization and dehumanization after restraint events to the patients [5, 6]. A recovery-oriented framework emphasising personal recovery “involves living as well as possible” in spite of any mental health issues [10], and includes maintaining hope during crises [10]. Within this framework, care providers may be more likely to consider the patient as a human being in their entirety, and consequently consider the patient to be jointly responsible for finding alternative approaches to restraints, based on the patient’s resources and former experiences. A humanizing care approach “provides eight philosophically informed dimensions of humanization, which together, form a framework that constitutes a comprehensive value base for considering both the potentially humanizing and dehumanizing elements in caring systems and interactions” [24]. We consider this approach might be useful to support care providers in preserving patients’ integrity and dignity, even if use of restraint becomes inevitable.

## Methods

To examine the body of knowledge on PIR s’, we carried out a scoping review following Arksey and O’Malley’s methodological framework constituting a five-stage approach. The scoping review proved to be suitable for defining and describing, as well as identifying practical implications, variations and experiences with PIRs’. Furthermore, it allowed for a broad approach to a topic of interest, as well as inclusion of studies regardless of their methodological design identifying research gaps and summarizing findings of research [25, 26].

### Stage 1: identifying research questions

Initially, we performed a broad search for PIR in the available scientific and professional literature, public documents and guidelines. After becoming familiar with the literature, we developed the three research questions to guide the review.

### Stage 2: identifying relevant studies

**Fig. 1:**
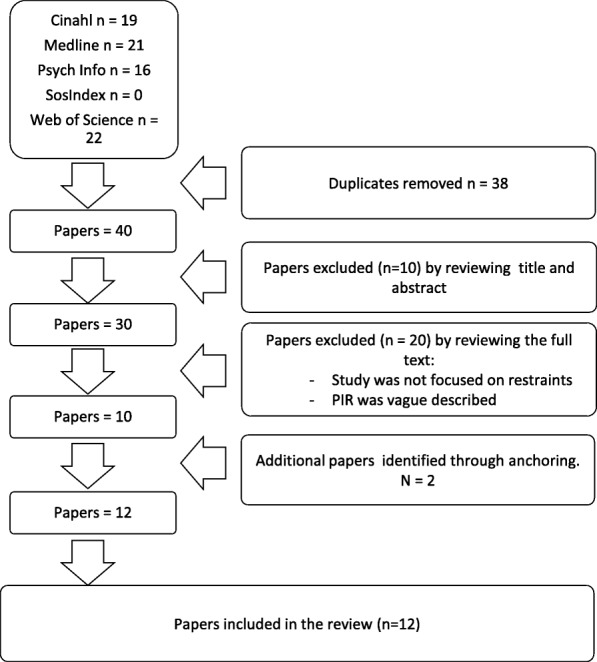
Study selection process

Systematic literature searches were carried out in September 2016 – May 2018 in five databases: Medline, PsychInfo, Cinahl, Sociological Abstracts and Web of Science. The search centred on three main concepts: 1) restraint; mechanical OR physical, AND 2) psychiatric OR mental, AND 3) post-incident review OR debriefing. The search terms, including Medical Subject Headings (MeSH terms) and synonyms for each of the main concepts were combined with OR. The search yielded 40 sources after duplicates were removed (see Fig. 1).

### Stage 3: study selection

**Fig. 2:**
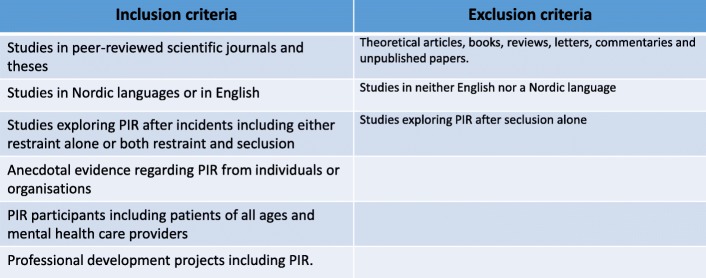
Overview over inclusion, − and exclusion criteria

The original aim of the review was to describe any available scientific knowledge on PIR after restraint alone, given that restraint and seclusion differ in terms of their legality and application, as well as their therapeutic and ethical consequences. In examining the literature, however, it quickly became clear that only a few publications fulfilled the criterion regarding restraint alone, so we changed the inclusion criteria in line with scoping review methodology [26]. The focus in this review will be on PIR s’ after restraint, even though some publications (*n* = 7) explore restraint and seclusion together. Figure 2 presents an overview over inclusion, − and exclusion criteria.

Two authors (U.E.H. and H.S.) independently reviewed all the abstracts and keywords using the inclusion and exclusion criteria. Any studies that were disagreed upon were further discussed, and a consensus was reached for all the articles included.

In addition, an ancestry approach was performed, reviewing and scrutinising reference lists from the retrieved full-text articles and review articles where other aspects of debriefing procedures were illuminated [22, 27] to detect any additional articles not identified in the computerised literature search [25]. This approach led to the inclusion of two more publications.

Arksey and O’Malley do not require a quality appraisal of the studies included in their review [25], but that approach is disputed [28]. In order to strengthen the quality of our review, we did choose to evaluate the studies.

The qualitative studies were evaluated following Polit and Beck [29]. Weaknesses in publications were identified, including a lack of theoretical integration and descriptions of the study population, analysis processes and risk of bias. Evaluation of the quantitative publications was based on narrative descriptions as none of the publications was comparable regarding design and outcome, so equal quality criteria could not be used.

### Stage 4: capturing the data

We employed an inductive approach in the analysis and synthesis of this review [30] Using NVivo 11 software [31], we carefully read the publications and examined their content related to the research questions. Study characteristics and manifest content, i.e. content close to the text [32] were identified regarding the first and second research questions, placed in a matrix and then compared for equality and differences.

### Stage 5: collating, summarising and reporting the results

The search for outcomes related to research question 3 that could deal with both measurable effects on S/R reduction and patients’ and care providers’ experiences consisted of two steps. The results from quantitative publications were ordered into a matrix for comparison. Experiences presented in qualitative publications were examined to identify common categories and, with some degree of interpretation, find themes across studies [30].

## Results

### Study characteristics

We included 12 empirical scientific studies in the review, including four qualitative studies [16, 33–35], six quantitative studies [21, 23, 36–39], and two studies using mixed methods [40, 41]. Only five publications reported on empirical research studies directly addressing of PIR [16, 23, 33, 40, 41]. The others described S/R reduction projects in which PIR were a component or an established or requested intervention between patients and care providers (thus implicitly described). Table 1 includes a description of the included publications.

**Table 1:** Description of the included publications

First author Date	Nation	Design/method	Aim	Setting and sample	Age group	Intervention
Petti 2001	United States	A combination data collection applying semi-structured interviews and a cross-sectional questionnaire on debriefing incidents	Explore role of PIR in a S/R reduction project	81 incidents, both patients and staff	Children and adolescents	Restraints and seclusion
Bonner 2002	United Kingdom	Descriptive pilot study Semi-structured interviews	Evaluate feasibility and helpfulness of PIR after restraints	Patients (*N* = 6) Staff (*N* = 12)	Adults	Restraints
Fisher 2003	United States	Cross-sectional study of patients and staff at clinic Observational design using questionnaire and register data from the clinic and the whole state (reference group)	Describe the results of a program to reduce S/R rates in a mental health hospital	Patients (*N* = 148; 25% response rate) Staff (*N* = 112; 15% response rate)	Adults	Restraints and seclusion
Ashcraft 2008	United States	Evaluation study with 58-month follow-up, implementing a new organisational program including PIR in two crisis clinics Registration of S/R rates	Reduce S/R use to zero S/R events	Two urban crisis centres, one small and one large	Adults	Restraints and seclusion
Bonner 2010	United Kingdom	Cross-sectional study assessing agreement on 6 statements (on a 7-point Likert scale)	Evaluate whether staff and patients found PIR helpful after restraint incidents	Patients (N = 30) Staff (*N* = 30)	Adults	Restraints
Azeem 2011	United States	Descriptive study using medical records reviewed over 33 months	Determine the effectiveness of six core strategies based on trauma-informed care at reducing S/R	Psychiatric hospital. Medical records (*N* = 458)	Children and adolescents	Restraints and seclusion
Azeem 2015	United States	Descriptive longitudinal study using register data on restraints incidents over 10 years at one clinic	Assess restraint reduction rates over 10 years in a clinic that implemented a restraint prevention programme	52-bed psychiatric hospital	Children and adolescents	Restraints
Lanthen 2015	Sweden	Descriptive design Interviews	Examine patients’ experience of mechanical restraints and describe the patient care received	Former psychiatric patients. (*N* = 10)	Adults	Restraints
Ling 2015	Canada	Descriptive study Audits of a sample of patient charts containing post-restraint event patient debrief forms	Examine PIR data to understand patients’ experiences before, during and after restraint events	Audits (*N* = 55)	Adults	Restraints
Riahi 2016	Canada	Retrospective register data study: registration of S/R episodes, number and average time over a 36-month evaluation period	Describe the process and value of implementing the six core strategies	Specialized, tertiary mental health care facility with 326 beds	Adolescents	Restraints and seclusion
Gustafs-son 2016	Sweden	Descriptive design Interviews	Describe nurses’ thoughts and experiences of using coercive measures during forensic psychiatric care	Nurses (*N* = 8)	Adults	All kinds of coercion
Goulet 2017	Canada	Pilot study with case study design Individual semi-structured interviews with patients and staff Pre-post study assessing the prevalence of seclusion and restraint before and after PIR	Evaluate a PIR intervention implemented in an acute psychiatric care unit	Interviews: Patients (N = 3) Staff (N = 12) Pre-post study: Anonymised administrative data (*N* = 195 admissions)	Adults	Restraints and seclusion

### How are PIR s’ defined and described?

Table 2 includes an overview of the results of research question 1. The term *PIR* is defined in two publications [33, 41], but descriptions of PIR indicate systematic intervention by using words as “rigorous problem solving”, “detailed behaviour analyses”, “chain analyses” etc. The purpose of conducting PIR was to learn how to prevent S/R through gentle, individual interventions such as talking or going for a walk and to identify and mitigate S/R-related patient harm.

**Table 2:** Definitions and descriptions of PIR

First author Date	Definitions	Descriptions		
		Purpose	Theoretical foundation or recommendations	Care philosophy
Petti 2001	Systematic debriefing	S/R reduction	Public recommendations	Strength-based care
Bonner 2002		After-incident support		
Fisher 2003	Detailed behaviour analysis	Mapping of patients’ and staffs’ views on S/R events and thereby S/R prevention	Public S/R reduction programme	Person-centred care
Ashcraft 2008	Chain analysis	Capturing of the viewpoints of patients who have experienced S/R	Public S/R reduction programme	Recovery-oriented care
Bonner 2010		Discussion of events at patients’ own pace in a nonthreatening way	NICE guidelines	
Azeem 2011	Rigorous problem solving	S/R prevention	Public S/R reduction programme	Trauma-informed and Strength-based care
Azeem 2015	Chain analysis of incidents	Restraint prevention	Public S/R reduction programme	Recovery-oriented, person-centred and strength-based care
Lanthen 2015			Quality and safety education for nurses project	Person-centred care
Ling 2015	‘an opportunity to talk about feelings, reactions, and circumstances surrounding an inpatient’s restraint experience, from the inpatient’s perspective’(p. 387)	‘an opportunity for clinicians to assess inpatients and determine necessary follow-up care’(p.387)	Public S/R reduction programme	
Riahi 2016	Formalised service-user debriefing	Exploration of events from patients’ perspectives to mitigate adverse S/R-related effects and use the lessons to inform future practice	Public S/R reduction programme	Recovery-oriented and trauma-informed care
Gustafsson 2016		Establishment of a communication forum for nurses and patients		
Goulet 2017	‘a complex intervention, taking place after an SR episode and targeting the patient and healthcare team to enhance the care experience and provide meaningful learning for the patient, staff, and organization’ [37]	Obtaining of patient feedback on their SR experiences	Bonner’s model (2008)	

Notes: Empty cells = not described

Two definitions of PIR s’ are related to both restraint and seclusion but vary on some points [33, 41]. Goulet and Larue define PIR as ‘a complex intervention, taking place after an SR episode and targeting the patient and healthcare team to enhance the care experience and provide meaningful learning for the patient, staff, and organisation’[41,p.212]. This definition indicates that PIR are learning tools not only for patients and care providers, but also organisations. Additionally, PIR s’ was usually based in public S/R reduction or quality improvement programmes. The stated treatment philosophies were (7 of 12 publications) strength-based, person-centred, trauma-informed and recovery-oriented.

### How are PIR s’ conducted?

The review showed that descriptions of how to conduct PIR s’ in practice varied in participants, timeframe, form and content of the conversation (Table 3).

**Table 3:** How is PIR conducted?

First author Date	Participants	Time	Content of PIR
Petti 2001	Nursing staff other than those directly involved with the incident	As soon as the patient can respond coherently to questions	Mapping of reasons for S/R, possible prevention actions and alternative measures
Bonner 2002	Patients and staff		Participants’ comprehension of what happened before, during and after the restraint event; mapping of needs for after-incident care
Fisher 2003	Patients and treatment team		Analysis of the events leading up to the S/R event and more long-term planning to avoid a repetition of S/R
Ashcraft 2008	Patients and staff		What patient and staff could have done differently and what staff could do in the future to prevent S/R
Bonner 2010	Staff, patients, caregivers and witnesses to incidents	Within 72 h	Mapping of the incident and surrounding events and consideration of what was helpful and unhelpful during the incident
Azeem 2011	Staff and patients involved	Within 48–72 h	Mapping of triggers, evaluation of interventions and possible S/R prevention alternatives and identification of traumatisation/retraumatization to patient and staff
Azeem 2015	Patients and staff involved in incidents, clinicians, physicians and sometimes hospital administrators	Within a few days	Analysis of the incident, triggers, helpful interventions and alternatives regarding S/R prevention
Lanthen 2015	Patients and staff Verbal and written follow-up		
Ling 2016	Verbal or written follow-up Participants are decided by the patient and the team	Within 24 h If an inpatient declines, new offer within 72 h	Patients’ feelings, reactions and circumstances regarding the restraint experience; mapping of needs for follow-up care
Gustafsson 2016	Patients and nurses who performed the coercive measure	“too much time’ should not have passed” [p. 41]	Exchange of reciprocal understandings of the S/R event
Riahi 2016	Patients and staff	As soon as possible after event is clinically indicated	Exploration of the event, identification of triggers, alternative options and identification and healing of restraint-related damage
Goulet 2017	Patients and staff members identified in the staff report	Within 24–48 h, but flexibility in practice	Review of events leading to the incident, factors involved, effect on patients and changes in future practice

Empty cells = not described

#### Participants

All publications, except one [16], defined the participants in PIR. In all the publications, patients and care providers participated in PIR, but the composition of care providers varied somewhat. The procedures involved participation by care providers who were both directly and not directly involved in the S/R incidents. Additionally, one procedure suggested including the treatment team, attending physician/psychiatrist and management representative [38]. In two of the most recent selected publications, inter-professional teams and patients decided with whom patients felt most comfortable meeting in PIR [33, 41].

#### Timeframe

Based on the time of conducting PIR, two approaches and procedures were described: first, within a timeframe expressed in hours; and second, when the patient was considered mentally capable of participating. Stakeholders’ viewpoints on the appropriate time were reported in two publications [35, 41]. One publication discussed patients’ viewpoints (*n* = 3) and proposed PIR 1 week after the SR episode; however, it was unclear whether the three patients agreed on the issue [41]. Care providers’ statements varied from asserting that PIR should be conducted within a certain timeframe to claiming ‘too much time must not have passed’ [35], or allowing wide variability in practice by minutes, hours, days and weeks [41]. Later care providers related this flexibility to when they considered the patients ready to talk about S/R and, in some cases, when the care providers themselves felt emotionally available. One publication referred: ‘With patients, you have to wait for the dust to settle, for yourself, but especially for them. If you do it the day after it’s like pushing a button and triggering something that hasn’t completely healed’ ([41], p.216).

### Form and content of the conversation

All the procedures described face-to-face meetings, while some procedures included a written evaluation in addition or as an alternative [33, 34]. Furthermore, descriptions of PIR emphasised a supportive, non-threatening atmosphere [23, 37, 40, 41].

Regarding PIR content itself, we found some differences in the procedures concerning questions for participants. All the procedures ensured that participants were asked about antecedents or triggers, any actual S/R incidents and possible alternatives for de-escalation in similar situations. Care providers were asked whether they could have handled the situation in another way, while that question was posed to patients in only three publications [33, 36, 41]. Finally, patients were asked about their emotional reactions in various ways, ranging from open-ended questions about feelings to direct questions about level of feelings, safety during procedures, maintenance of privacy and dignity [16, 33, 34, 36, 41]. Patients could thus express the need for after-incident care. One publication referred to the possibility of using PIR as a tool for the mutual sharing of emotions between patients and care providers, with the aim of opening a dialogue that ‘perhaps creates an even stronger bond of trust between patients and nurses’ ([41], p.216).

### Experiences of PIR

The experiences of PIR described in the articles included 1) measuring the outcome of S/R reduction connected to the implementation of programmes including PIR (quantitative results) and 2) stakeholders’ descriptions of their experiences of participating in PIR (mostly qualitative results).

#### Reduction of S/R

As shown in Table 2, we found that PIR was part of S/R prevention programmes in six publications. The programmes were implemented in different institutions from acute clinics to tertiary hospitals, and the patients were children, adolescents and adults. One publication reported results from a pilot project, implementing PIR as a single intervention alone [41].

As well, no studies were randomised, but some formed control and reference groups in various ways. All the studies measured the outcomes in different ways, so it was not possible to pool the results into a meta-analysis. S/R reduction was measured in two ways: 1) reduction in the number of episodes and 2) in the duration of episodes. The results are presented as follows.

The implemented programmes including PIR contributed to significantly reducing S/R episodes [21, 36–39, 41]. Fisher [21] found a 67% decline in S/R rates when using their clinic’s history data on S/R events, making the clinic their control. In addition, this clinic went from a S/R event rate 46% higher than the state average to 44% lower, using state reference data on S/R events as controls. Another study made its desired outcome no S/R incidents in 1 month. Ashcraft and Anthony [36] implemented an organisational S/R reduction programme in two clinics and continued the programme until that goal was achieved, which took 10 months regarding seclusion and 2 months regarding restraints at the small centre and 31 months regarding seclusion and 15 months regarding restraints at the large centre. Azeem et al. [37] compared the first 6 months to the last 6 months of a study period where care providers were trained in the six core strategies. Seclusion and restraint data showed 93 incidents involving 22 patients (mean 4, 2 incidents/patient) in the first 6 months versus 31 incidents involving 11 patients (mean 2, 8 incidents/patient) in the last 6 months. Another study of Azeem et al. [38] took a 10-year perspective on the programme implementation. Mechanical restraint incidents fell from 485 in 2005 to 0 in 2014, with no events in the past 3 years. Physical restraint incidents decreased by 88%, from 3033 in 2005 to 379 in 2014 [35]. Decreased duration of S/R episodes was reported in three articles [21, 39, 41]. Fisher [21] found that the duration of S/R decreased by 92% when examining their clinic’s historical data on S/R events. Riahi, et al. [39] found the average length of a mechanical restraint or seclusion incident decreased 38.9% over the 36-month evaluation period. Goulet, et al. [41] reported reduced use of seclusion, not restraint, while the median time spent in seclusion, but not restraint, decreased significantly from pre- to post-PIR.

#### Stakeholders’ experiences of participating in PIR

Both patients and care providers reported that PIR helped promote recovery processes [34, 35, 41]. Care providers reported that PIR contributed to increased professional reflexivity, which in turn resulted in improved patient care. They also appreciated that PIR provided an opportunity to review the restraint incident.

Bonner and Wellmann [23] evaluated whether patients and care providers found PIR helpful after restraint events. A majority of the patients (*n* = 30) and care providers (n = 30) who responded to a six-question post-incident survey considered PIR helpful after restraint events ([23], p.38–39), except that 61% of care providers and 20% of the patients believed that the restraint incident could have been predicted. Risk of bias is discussed in the Bonner and Wellmann’s study as all the 60 informants participated in the study [23].

### Recovery promotion

Recovery promotion emerged as a theme through both patients’ participation in PIR and in further issues discussed in PIR [23, 33–36, 41]. By participating in PIR, patients may have been empowered by contributing to recovery-promoting alternatives to S/R. For example, in one publication a care provider expressed; ‘We have to find ways to prevent this from happening again. What can you do? What can we do? If you want to avoid this, if you want to find ways not to relapse, we have to talk about it’ [41]. From care providers’ perspective, PIR had the potential to strengthen the patients’ identity: ‘He seemed satisfied and proud to have been able to express himself and be heard’ [41]. Regarding care providers’ experiences, the majority of patients claimed that PIR gave them an opportunity to review restraint events they would not otherwise have had [23]. Additionally, PIR seemed to provide a way for the patients to process and stimulate an understanding of the situation by talking about it [23, 34, 41], with the aim to promote hope and connectedness. Former patients in Lanthen’s study [34] considered adapting to restraint-related trauma as essential, allowing them to move on from the experience and continue their personal recovery processes.

### Increased professional reflexivity

In Bonner and Wellman’s study, nearly all the care providers claimed that PIR was useful for reviewing incidents of restraint and offered an opportunity to look over restraint events they would not have otherwise [23]. In chain analysis of S/R events, the patients reported S/R causes, care providers’ incident management, emotions before, during and after the incident and alternative measures for future S/R events [16, 33, 34, 36, 40, 41]. The antecedents to the S/R events could be hospital and ward-level factors, such as disturbed wards, miscommunication, patients’ unmet needs, conflicts between patients and care providers and patients’ lack of autonomy. Further, PIR revealed that S/R incidents caused strong negative feelings among patients, who described S/R as unnecessary and punitive, fuelling anger, sadness and resentment [33, 34, 40]. In addition, S/R was related to traumatisation and re-traumatisation and damaged relations between care providers and patients [16, 33, 39, 41]. Patients and care providers who participated in PIR reflected on how care providers could meet patients’ individual needs before and during S/R events by implementing alternative interventions.

A care provider expressed; “we bring some of our experience. New people bring new ideas too, so I think combining them together, you try to see what you can do better with everyone’s ideas” [41]. Possible alternatives were then recorded in patients’ care plans so that mitigating efforts could be implemented immediately. This individualised approach seemed to de-escalate situations, possibly helping to prevent S/R incidents [33, 34, 36, 38, 40]. Additionally, publications reported that information from PIR led to changes in organisations, but it was not always clear how these changes emerged, as they were not further described [36, 38, 40]. For example, ‘perhaps the most important implication of this study is to underscore the importance of debriefing as an indicator for continuing to introduce and track elements representing cultural challenges in this organization’ ([40], p.124).

### Processing the incidence

Benefits for care providers were mentioned in two publications [35, 41]. Goulet et al. [41] reported that PIR not only raised awareness about the trauma experienced by patients but also helped care providers manage their own feelings. Gustafsson and Salzmann-Erikson [35] argued that systematic PIR improved the working conditions of nurses participating in coercive measures by reducing stress. In addition, nurses [35, 41] viewed PIR as a way to restore trust relationships, but we did not find any patients who said the same.

## Discussion

The review shows that scientific knowledge on PIR is limited and the studies vary in quality and design. Furthermore, evaluations of S/R reduction programs are often based in local, ideal-driven development work in practice, with limited resources to conduct systematic outcome studies and without the involvement of any larger research environment or external perspectives. These studies lack some of the rigorous design provided by, for instance, experimental design. We, therefore, cannot conclude that PIR as an individual intervention contributes to S/R reduction even though Goulet’s pilot study [41] gave positive results according to seclusion. Nevertheless, S/R reduction programs we consider to be non-experimental programs developed in practice and seem to be largely effective, increasing the importance of a need for high-quality intervention research in this field of practice. Still, these methodological limitations mean that so far, we not can draw a solid overall conclusion on efficacy and, therefore, cannot recommend PIR as a mandatory procedure for S/R reduction alone.

Despite the lack of evidence for PIR contributing to S/R reduction, the results in this review indicates a contribution from PIR nevertheless. PIR could promote recovery and increase professional reflexivity, leading to improved care. These important indications are elaborated further in the following sections.

### Potential of PIR for patients’ personal recovery processes

The results of this review point to PIR as an effective intervention for mitigating S/R-related harm. Therefore, we believe it is relevant to discuss the results in terms of a recovery-oriented framework and a humanising care approach to nursing and ethics (10, 24). PIR represent an arena for the patient to regain status lost during the S/R event. Subject status will be an assumption for patients’ active participation and engagement in planning of treatment and care [10].

According to Buber, a “Subject–Subject/I–Thou dialogue” [42] can establish “a world of relation” [42], between persons. In the context of PIR, a Subject–Subject relationship between patient and care provider is optimal, even though, in the case of mental health services providers interacting with patients, there will always be an imbalance of power between stakeholders. However, an approximate Subject–Subject relationship might be preferable to an I–It relationship [42] and support the CHIME recovery processes of Connectedness, Hope, Identity, Meaning and Empowerment, processes that are significant for personal recovery [10, 43].

Patients’ expressed views on antecedents and triggers when participating in formulating care and crisis plans might promote recovery through agency and empowerment. [10, 44]. In addition, asking patients if *they* could have acted differently [34, 36, 41] minimises their loss of personal responsibility during crises, a central value in recovery-based care [10].

Within a framework of humanising care, PIR has the potential to contribute to patients’ re-humanisation after S/R-related emotions that can be experienced as dehumanising [6] as PIR can facilitate togetherness, uniqueness and sense-making [24]. These dimensions are compatible with the CHIME processes. Furthermore, PIR s’ provide a forum where sense-making can occur, if care providers give patients information and explain assessments for S/R use. By getting an explanation, the patients may perceive that care providers applied the ethical principles of proportionality and purposefulness and their intentions were influenced by beneficence. Consequently, being treated like a human being can lead to patients perceiving the restraint less negatively [45].

Regarding the conflicting results from debriefing studies [20], the descriptions of PIR in the selected publications indicate planned and structured dialogues with focus on the chain analyses of the S/R incident, but with minor focus on emotions (Table 2).

From an emotion-regulation perspective, constructive, insightful and controlled processes after emotional episodes lead to positive outcomes and create opportunities to re-evaluate events, thereby supporting identity regulation, which is central in recovery processes [10, 46]. In addition, since both patients and their mental conditions vary, PIR content related to sharing emotions must be take a person-centred approach [10].

In line with a recovery-based framework, a patient’s voice must be heard when it comes to PIRs’ timing as well as which participants should be included in the PIR.

Therefore, ‘the golden time’ [35] for PIR is essential; doing it early can violate patients’ integrity and uniqueness and contribute to dehumanisation through homogenisation and the loss of the personal journey [24]. However, waiting too long can increase negative feelings in patients, such as isolation and loss of meaning [34, 35].

Studies show an imbalance in PIR in terms of representing patients and care providers’ voices, with care providers clearly in the majority. In two studies [33, 41], patients have some influence over which staff members participate. In other studies, the PIR procedure itself determines the participants. Thus, the system has the advantage over the patients, since they are in a dependent and usually powerless position [47]. To address this imbalance in representation, it may be helpful to invite a trusted person to participate in PIR, for example, the patient’s next of kin, a supportive peer or an advocate [10, 48].

However, we did not find this alternative in the articles. Conducting PIR with care providers whom patients trust aligns with a recovery-based approach, but we will claim that care providers’ perspectives may be unclear or lost if participating care providers in PIR were not present during the S/R incident.

Conducting PIR in a supportive and non-threatening attitude [23, 37, 40, 41] is in line with an atmosphere characterised by human values, which can be crucial to patients’ psychological and moral perceptions of coercion in care in general [45]. In the case of PIR, care providers whose attitude is characterised by respect and who appear to be flexible, trusting, friendly and oriented towards collaboration on ideally equal terms might confirm patients as persons by promoting patients’ “insiderness” [49]. Patients’ well-being and identity might then be strengthened, thereby constituting caring power, the opposite of consequences of detached care [49]. PIR’s potential to restore the therapeutic relationship damaged in S/R interventions was described in two publications [33, 41], but this possibility was not presented from the patients’ perspective. This issue needs more exploration taking into consideration patients’ views.

### Potential of PIR for care providers’ reflection on action and processing

Reflection is considered to be an essential quality in knowledge production and professional development [50, 51]. In results, care providers see PIR as an arena for learning by reflection on action [50] that involves reflecting on how attitudes and caring practices can change. This reflection may be useful in potential future S/R-related situations as it provides an extended repertoire of alternative reflection-in-action measures for reflecting on an incident while still benefitting the situation at hand, rather than simply reflecting on how to act differently in the future [50]. Although based on the literature we cannot conclude that PIR contribute to S/R reduction, we can highlight the potential for care providers’ learning through reflection on action with patients. This reflection has the potential to promote the moral elements of care, such as attentiveness, responsibility, competence and responsiveness, and thereby improve the quality of care [52].

Furthermore, PIR gives care providers an opportunity to process S/R incidents that might create mental strain for them as professionals, although S/R-related damage to patients and strain to care providers should not be viewed as equivalent due to the power imbalance. Processing can help care providers deal with emotional and moral distress if they view restraint events as morally uneasy [53, 54]. Doing so might improve their ethical and professional care as care providers ‘in touch with and guided by their values are more likely to feel inspired and empowered’ [55]. The described sharing of emotions between patients and care providers [41] might be professionally controversial. However, from the recovery perspective, sharing emotions might support patients’ personal recovery processes [10] if care providers do not treat PIR as an arena for their personal debriefing.

### Potential of PIR for organizational development

Previous research showed that a number of perspectives regarding S/R reduction, among them workforce development, need to be studied [10, 13]. Creating reflexive cultures, therefore, is important in addressing coercive practices, including systemic and cultural concerns [54]. As shown in the results (Table 2), PIR is often implemented in organizations with defined care philosophies based on human values that provide alternatives to deficit-based medical models by understanding deficits within broader contexts [10, 55]. These care philosophies emphasize user participation, viewing patients as experts along with care providers. In the perspective of evidence-based healthcare, aggression and agitation are related to patients’ diagnosis and symptoms, suggesting that care providers perform certain actions independent of the context. The disparity between patients and care providers in Bonner and Wellmann’s study regarding prediction of restraint incidents [23] may reflect a more optimistic attitude from care providers than from patients. That might indicate different frame of reference and thus, different expectations and solutions.

In recovery-based healthcare, however, patients’ and care providers’ reflections and ongoing dialogues on the antecedents and triggers of restraint events and inclusive environmental factors, may contribute to organisational development and care improvement, as reported in the results and supported by Goulet and Larue’s definition of the debriefing procedure [22, 41]. Relevant improvement issues can include care providers’ educational needs and patients’ expressed needs for more supportive ward environments, and by that support patients’ wellbeing [56].

Legislation, as in Norway and Denmark [3, 57], or guidelines as in United Kingdom and some states in USA [58, 59], raises the question of degree to which PIR should be standardised versus conducted in a flexible manner. PIR as a strict procedure might increase PIR’s feasibility and care providers’ safety when conducting an often-demanding dialogue. In a manual-based treatment organization however, PIR might be another manual to check off, risking minimizing the documented benefits of PIR reported in this review, while additionally increasing the risk of objectifying patients. The reported differences in carrying out PIR therefore, indicate that PIR cannot follow strict procedures as in a manual. Instead, PIR should be conducted in accordance with a recovery-based philosophy [10] that gives care providers the flexibility to individualise assessments regarding timeframes, participants and content.

Consequently, in addition to reflexive professional practice, care providers need to be ethically mindful and sensitive to ethically important moments in everyday practice, acknowledging them as significant [60]. In a recovery perspective, then, PIR should not be implemented as a separate procedure within organisations, but should be integrated with ethical issues, treatment philosophies, quality improvement and service development [61].

### Limitations and strengths of the review

A strength of this review is that it examines a knowledge base in an area rarely explored despite professional and political guidelines recommending PIR. Another strength is the comprehensive, systematic search strategy supported by a qualified librarian and the examination of relevant reviews in both the scientific and the grey literature. According to Arksey and O’Malley [25], quality assessment of the included publications is not necessary, but we consider our narrative description of the quality of the selected publications to be a strength as methodological shortcomings affect the quality of findings.

One limitation was the lack of publications explicitly examining PIR, so the inclusion criteria were changed to articles exploring PIR after restraint alone and articles exploring PIR after restraint and seclusion together. As described, variations in the studies’ design and quality required appraising and determining which studies to include. In addition, we could have missed relevant information by excluding reports published in local, non-indexed journals and books. Consultations by practitioners and patients/consumers were not included but could have produced more nuanced results [25]. We address this issue in a separate project.

## Conclusion

This review of scientific literature presents PIR as an intervention with the potential to benefit patients’ recovery processes, care providers’ reflection on action, processing and organisational development. In sum, PIR seems to be promising for restraint (R) prevention and the promotion of a more professional, reflexive, ethical care culture in mental health services. To achieve these outcomes, PIR should be implemented in supportive environments with care philosophies based on human values and care providers’ ethical mindfulness.

The recovery and humanising care approach seems to offer opportunity to prevent and process restraint events, thanks to its focus on patients’ individual needs. However, its overall application needs to be further explored. In addition, it would be beneficial to further examine stakeholders’ experiences of PIRs’, and take into account both patients’ and care providers’ perspectives. The patients’ dependence on the system, especially when being compulsorily detained, can however be critical to their participation in PIRs’. Thus, this issue needs to be addressed.

In both scientific studies and in society, patients’ voices on the consequences of coercion and care improvement are underrepresented. This lack conflicts with ‘the moral claim to call attention to the necessity of honest inclusion of everyone’s perspectives in a democratic society where caring is highly participatory’ [62].

## Abbreviations

CHIME: Five recovery processes that are significant for personal recovery: Connectedness, Hope and optimism about the future, Identity, Meaning in life and Empowerment.
PIR: Post incident review
